# Second harmonic generation of optical spin−orbit interactions in hybrid plasmonic nanocircuits

**DOI:** 10.1515/nanoph-2024-0725

**Published:** 2025-03-20

**Authors:** Junjun Shi, Kangcheng Jing, Li Li, Wenjun Zhang, Tianzhu Zhang, Xiaobo He

**Affiliations:** Shandong Provincial Engineering and Technical Center of Light Manipulations & Shandong Provincial Key Laboratory of Optics and Photonic Device, School of Physics and Electronics, Shandong Normal University, Jinan 250014, China; Institute of Physics, Henan Academy of Sciences, Zhengzhou 450046, China; Henan Key Laboratory of Quantum Materials and Quantum Energy, School of Quantum Information Future Technology, Henan University, Kaifeng 475001, China

**Keywords:** second harmonic generation, spin–orbit interaction, surface plasmon, CdSe nanowire, hybrid waveguide

## Abstract

The manipulation of nonlinear spin–orbit interaction at the nanoscale is crucial for advancing information processing in integrated nanophotonics. However, the weak spin–orbit interaction (SOI) in conventional waveguide materials significantly limits the efficiency of nonlinear optical processes. In this work, we design a hybrid plasmonic waveguide composed of a gold film and a Y-branch CdSe nanowire, which addresses the aforementioned limitations. The designed hybrid structure enables efficient directional emission of second-harmonic generation (SHG) via control of the polarization of the excitation light. The transversely emitted SHG can be visualized for directly imaging the SOI. Our work not only provides a way to enhances the efficiency of the nonlinear SOI but also a promising platform for further advances in integrated photonics and nonlinear optics.

## 1 Introduction

Light has intrinsic spin angular momentum (SAM) and orbital angular momentum (OAM) [1]. The spin–orbital interaction (SOI) resulting from light–matter interactions leads to fascinating phenomena, such as the optical spin hall effect [2], [3] and spin-momentum locking [4]. SOI has attracted significant interest because of its potential applications and has led to noteworthy advancements in selective routing [5], [6], [7], [8], information processing [9], and chiroptical detection [10], [11] on chip. In general, the conversion of SAM to OAM results in a greater number of spin–orbit optical states for the nonlinear field than that for linear field owing to the interplay between the linear spin–orbit coupling and the nonlinear process [12]. The polarization state of an input can be transferred to the OAM of the nonlinear field via interaction with the vortex input [13]. Moreover, vortex second harmonic generation (SHG) can be excited by circularly polarized Gaussian beams via SOI [14], [15]. However, advances in these fields are limited primarily by the weak SOI at the nanoscale and the fact that most circuits operate in the linear optics regime. Therefore, efficiently extending SOI into nonlinear regimes is necessary for multiplexing and achieving a greater number of on-chip operational frequencies.

Surface plasmons, which are a hybrid state of collective oscillations of electrons and photons, feature small mode area and giant local field enhancement. Hence, surface plasmons can be exploited to significantly enhance light–matter interaction in the subwavelength regime [16]. The strong SOI in the plasmonic structure, including in metal particles [17], branched gold nanowires [18], and plasmonic slot waveguides [10], [19], has been confirmed in previous studies. However, the nonlinear response of the metal (such as Au and Ag) originates primarily from the surface [20], and the large ohmic loss of the surface plasmon leads to a short propagation length [21]; this, in turn, results in weak nonlinear response and consequently, poor nonlinear circuit performance [22]. Integrating dielectric materials with large nonlinear susceptibility onto the plasmonic structure can address this limitation [23], [24]. Compared to pristine circuits, a hybrid circuit leads not only to enhanced SHG but also to a high routing ratio [25]. However, in such hybrid circuits, the mismatch in alignment between the component of the nonlinear susceptibility and the maximum local field weakens the nonlinear response [26].

In this study, we demonstrate spin–orbit controlled SHG in a hybrid plasmonic nanocircuit that comprises a Y-branch CdSe nanowire and a gold film separated by a 5 nm Al_2_O_3_ layer. We achieve an SHG conversion efficiency of up to 3 × 10^−6^ W^−1^ by exploiting the large nonlinear susceptibility of CdSe and the local field of surface plasmon polaritons (SPPs). The experimental results as well as the numerical simulations show that the hybrid plasmonic waveguide can selectively couple and directionally route the fundamental wave (FW). The beam-splitting ratio depends on the spin state of the excitation. Moreover, SHG also exhibits evident spin-controlled unidirectional power flow upon varying the incident polarization, which is in good agreement with numerical predictions. The transversely emitted SHG generated by the interaction between the counter-propagating FW modes can be directly visualized, which provides a simple means for directly imaging the SOI compared with the scanning near-field optical microscopy [27]. The results obtained in this study offer novel possibilities for nonlinear chiral nanophotonic applications.

**Figure 1:**
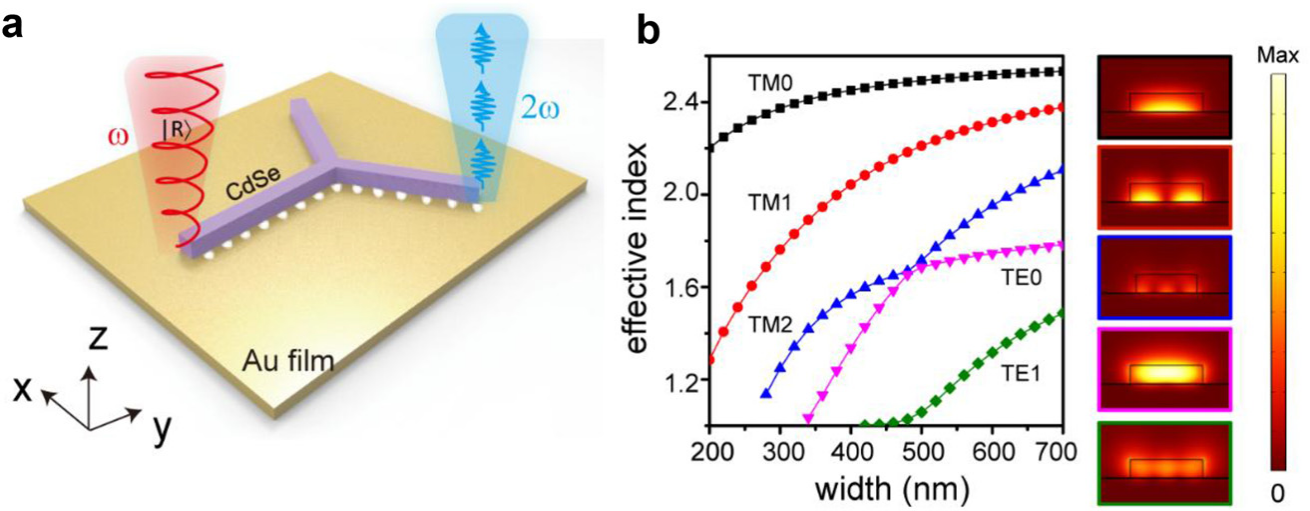
Design of the branched hybrid plasmonic waveguide. (a) Schematic of spin–orbit controlled SHG in a hybrid plasmonic waveguide. (b) The effective refractive index and corresponding electric field distribution of the TM_0_ (black), TM_1_ (red), TM_2_ (blue), TE_0_ (purple), and TE_1_ (green) modes as a function of the width of the CdSe nanowire. In the simulations, the thickness was set to 150 nm, and the wavelength was 800 nm.

To achieve spin-controlled SHG, we employed a typical branched hybrid waveguide structure consisting of a CdSe nanowire placed on a Au mirror, with a 5 nm Al_2_O_3_ insulating layer in the gap between them (Figure 1a). Before the experiment, we conducted mode analysis using the finite element method. In the simulations, the width of the CdSe nanowire was varied from 400 to 700 nm, and the thickness was set to 150 nm. Figure 1b shows the four lower modes supported by the hybrid waveguide. The profiles clearly indicate that the TM mode is an SPP-like mode because the electric field distribution is predominant near the Au surface. This indicates that both the TM_0_ and TM_1_ modes can be efficiently excited when the polarization of the incident light is parallel to the nanowire (Figure 1b). Because the size of the CdSe nanowire is larger than the typical size of the diffraction limit at the wavelength of 800 nm, the waveguide could also support photonics modes, such as the TE modes. In particular, the parity of the TM_1_ mode is distinct from those of the other three modes. When both TM_1_ and other modes are excited by circularly polarized light, the SPPs exhibit a periodic chiral pattern [28]. The period Λ is inversely proportional to Δ*n* (the difference between the effective refractive indices of the two modes) and can be calculated using Λ = *λ*/Δ*n*, where *λ* denotes the wavelength. To effectively guide the SPPs, the length of the main CdSe nanowire must satisfy $L=\left(m+\frac{1}{2}\right)$ Λ (*m* is an integral) to ensure an asymmetric field distribution at the junction.

**Figure 2:**
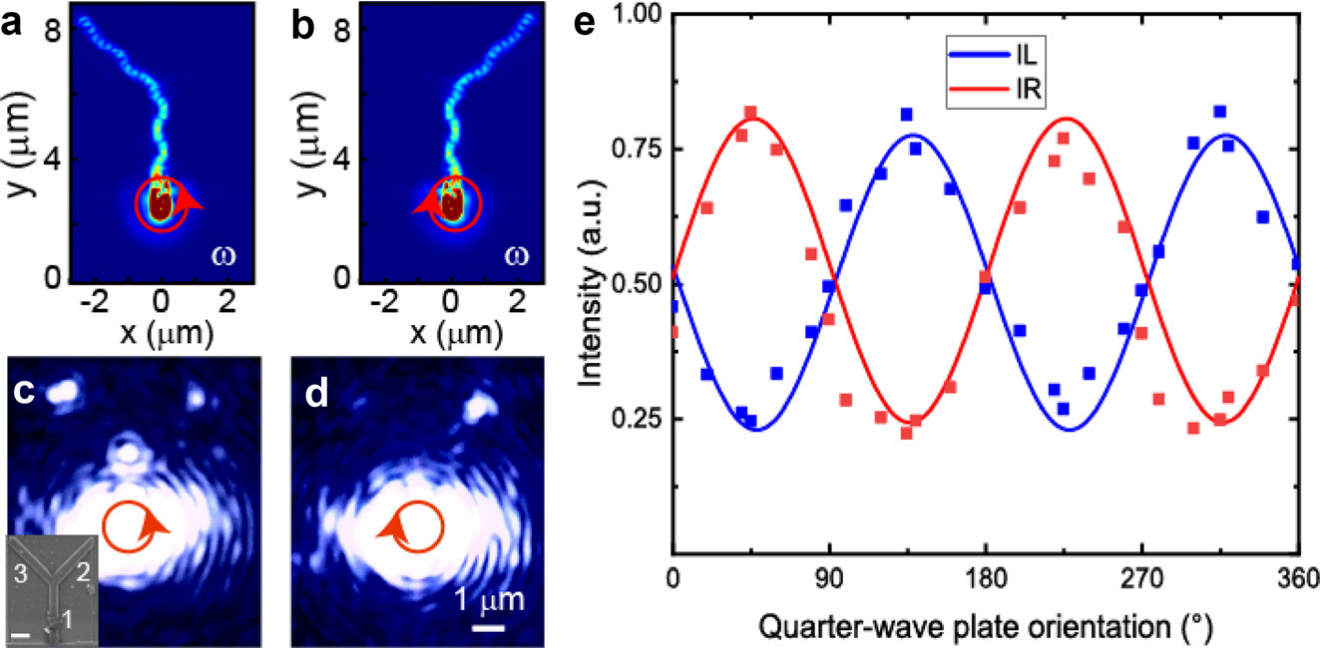
Spin-dependent directional propagation of SPPs in a hybrid branched nanowire. (a, b) Simulated electric field distribution under LCP (a) and RCP (b) illumination. (c, d) Experimental false-color image under LCP (c) and RCP (d) illumination. The inset shows a scanning electron microscopy image, and the scale bar is 1 μm; the numbers 1, 2, and 3 denote the branches. (e) Simulated (solid lines) and experimental (squares) values of the emission intensity from the left (blue) and right (red) terminals as a function of the quarter-wave plate orientation.

The strong SOI at the wavelength of 800 nm was numerically validated via finite difference time-domain simulations (Section 3.1). In the calculations, the width and thickness of the CdSe nanowire were set to 500 and 150 nm, respectively. According to the results shown in Figure 1b, to match *L* with the Λ, the intersection was set at a distance of 3.5 μm from the input port. When the left or right circularly polarized (LCP or RCP) beam was normally illuminated onto the port, the light displayed completely different propagation directions and a pronounced complementary twisted orbital momentum density flow in the near-field (Figure 2a and b). The routing ratio varied with the polarization state. Interference was observed around the terminal owing to the large loss of the SPPs and reflection from the terminal. A waveguide was fabricated via focused ion beam (FIB) milling for experimental demonstration of SOI-controlled SPPs (see the Section 3.2 for further details**)**. As shown in Figure 2c, the widths of the No. 1, 2, and 3 branches were approximately 520, 616, and 539 nm, respectively. The thickness was ∼150 nm, as indicated by the atomic force microscope image shown in Figure S1. A custom-built microscope was used to investigate the optical response of the waveguide at different excitations **(** Section 3.3 **)**. The differences in the intensity distributions at the two terminals for the circularly polarized beam demonstrate that the results are in good agreement with the simulations (Figure 2c and d). The minimal scattering from the middle of the CdSe waveguide affirms the ability of the waveguide to guide light. Spin-controlled emission was achieved by varying the quarter-wave plate orientation. The maximum routing ratio was merely 1:3.3 (Figure 2e), which is smaller than that reported in the literature [10], [11]. The simulations reveal that the directional routing in Y-branch nanocircuit results from participation of the five modes (Supporting Information S4). Our hybrid waveguide exhibits adequate circuit performance under the FW regime (Supporting Information S7).

**Figure 3:**
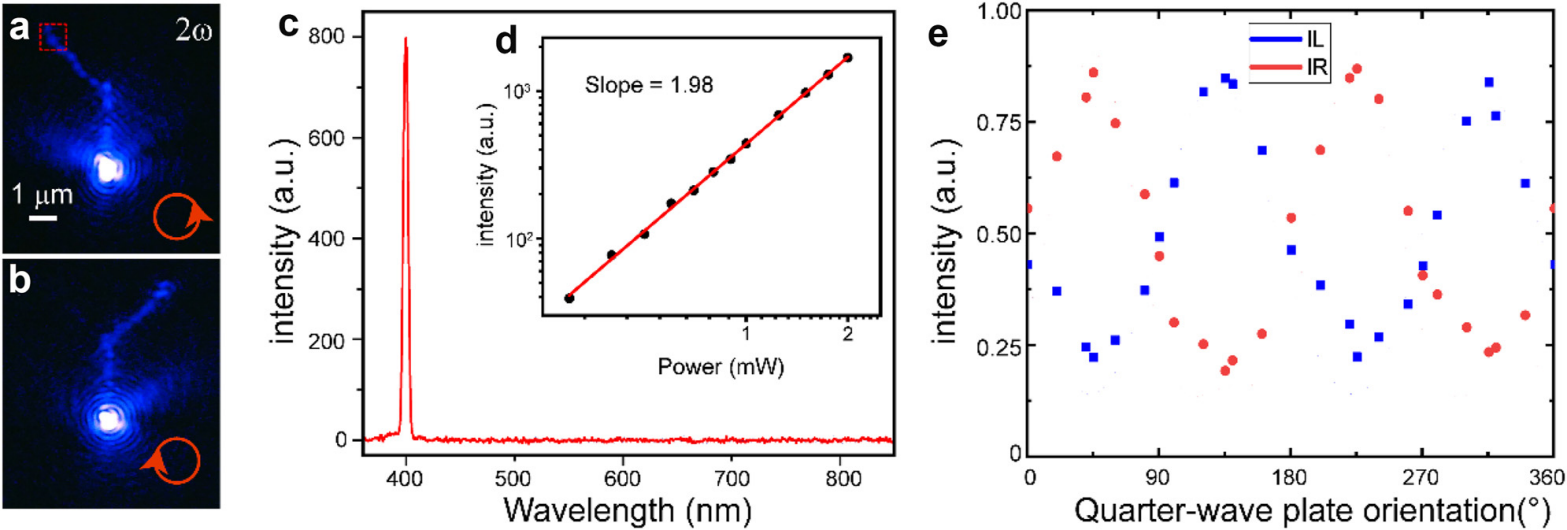
Spin-controlled SHG in a hybrid branched nanowire. (a, b) Experimental false-color SHG image under LCP (a) and RCP (b) illumination. The scale bar is 1 μm. (c) Representative SHG spectrum from the region marked by a red rectangle in panel (a). (d) The power dependence of SHG. (e) Experimental SHG emission intensity from the two terminals as a function of the quarter-wave plate orientation.

Next, we examine in detail the nonlinear response of the circuit. To demonstrate spin-controlled SHG, a bandpass filter was inserted into the collection path to remove the FW. At the excitation wavelength of 800 nm, the images shown in Figure 3a and b were obtained when circular-polarized light was normally focused onto the port. The representative spectrum collected from the terminal is shown in Figure 3c. The signal at 400 nm exhibited a good quadratic response (Figure 3d), confirming SHG. An SH conversion efficiency as high as 3 × 10^−6^ W^−1^ can be achieved via a combination of the high nonlinear coefficients of CdSe and large field enhancement of SPPs (Supporting Information S6). As expected, the SHG exhibits the similar spin-controlled emission as the FW. However, the number of spatial trajectories for SHG was apparently higher on the output branch waveguide compared to that for the FW. Apart from the twisted flow, the trajectories exhibited another period that was considerably shorter than Λ. We attribute this unique SHG distribution to the interaction between the counter-propagating FW–FW modes. The main reasons are as follows. On the one hand, the large loss of the plasmons at 400 nm cannot lead to such large propagation lengths; this rules out the possibility that the phase match or phase mismatch leads to periodic SHG emission [29]. On the other hand, the SH image is similar to the near-field distribution of the FW, and both the FW and SHG exhibit twisted flow with an interference pattern around the terminal (Figures 2a and 3a). The interaction between the counter-propagating FW modes, resulting from the counter-propagating FW reflected at the terminal, leads to transversely divergent SHG with an interference pattern, and the period of the interference pattern is determined by the Δ*n* between the counter-propagating FW modes (Figure S4) [30]. The SHG Fourier image (Figure S5), wherein the SHG emission is in the plane perpendicular to the branch waveguide, confirms this assertion. The maximal routing ratio of 1:4.3 **(** Figure 3e **)**, which is marginally higher than that of the FW **(** Figure 2e **)**, was achieved by rotating the quarter-wave plate. The intensity for SHG was quadratically proportional to the excitation, which indicates that the routing ratio of SHG must be approximately 1:10. This large deviation may be attributed to the inconsistency in the SHG and FW emission angles.

## 2 Summary

In conclusion, we have proposed an efficient spin-controlled SHG and FW in a hybrid plasmonic circuit. The SHG conversion efficiency reaches 3 × 10^−6^ W^−1^ owing to the large susceptibility of CdSe and the giant local field of SPPs. An analysis of the SHG trajectories indicates that such spin-controlled SHG results from the nonlinear interaction between the counter-propagating FW modes, and the zigzag pattern is caused by the interference of co-propagating FW. A simple control over the polarization states of the excitation promotes approach to the SHG routing direction. The routing ratio for SHG is marginally higher than that for the FW. Our results not only enable spin-controlled channels in both the FW and SH regimes but also help in visual on-chip characterization of the polarization state and SOI [27]. Our work paves the way for integrated versatile nanophotonic circuits by taking advantage of the hybrid plasmonic waveguide [31], [32].

## 3 Methods

### 3.1 Simulations

The simulation results presented in this paper were obtained using the finite-difference time-domain method with perfectly matched layer boundary conditions to absorb the scattered electromagnetic radiations. The waveguide was composed of CdSe, and the optical constants of gold were based on the experimental data reported by Johnson and Christy. Convergence tests were performed to ensure that meshing and boundaries had minimal impact on the solution. Circular polarization was obtained using two 4-μm-diameter linearly polarized coherent Gaussian beam sources that were polarized orthogonally with a phase difference of 90°. Linear-polarized, LCP, and RCP light was obtained by varying the polarization angle. The width and height of the CdSe waveguide were set to 500 and 150 nm, respectively, to reduce propagation loss while maintaining moderate mode confinement.

### 3.2 Sample fabrication

First, 200 nm Au was deposited on the Si wafer via thermal evaporation (JSD-400, JIASHUO Vacuum Technology) at a rate of 0.5 A/s, and an ultra-smooth gold film was then peeled off from the wafer using a UV curing adhesive (Norland optical adhesive 61). Next, 5 nm of Al_2_O_3_ was deposited on the ultra-smooth Au film via atomic layer deposition. CdSe nanoribbons with a width of approximately 8 μm, a length of approximately 20 μm, and a thickness of approximately 150 nm were then obtained via chemical vapor deposition. Thereafter, the CdSe nanoribbons were precisely transferred onto the ultra-smooth Au film via mechanical transfer. Subsequently, the Y-branch CdSe waveguide structure was etched on the nanoribbons via FIB milling to complete the sample preparation.

### 3.3 Experimental setup

A collimated beam of femtosecond laser was focused onto the sample surface using a 100× microscope objective (NA = 0.9, Olympus). The polarization of the incident laser beam was controlled by inserting a polarizer and a quarter-wave plate in the input optical path. The polarization state of the incident beam could be continuously switched between LCP, LP, and RCP by rotating the quarter-wave plate. We used two bandpass filters (FF01-380-420, Semrock) to remove the fundamental light in the SHG collection path. The SHG signal was directed to either the spectrometer (iHR 3200, Horiba Jobin Yvon) for spectroscopy or to the CCD camera (Retiga 3000, QImaging) for imaging.

## Supplementary Material

Supplementary Material Details
