# Supplementary material for: Second harmonic generation of optical spin−orbit interactions in hybrid plasmonic nanocircuits
Source: Nanophotonics. 2025 Mar 20;14(7):1003–7. doi: 10.1515/nanoph-2024-0725 (PMC11980878; doi:10.1515/nanoph-2024-0725)
Supplement: Supplementary file 1 — Supplementary Material Details [file j_nanoph-2024-0725_suppl_001.docx]

**Second harmonic generation of Optical Spin−Orbit Interactions in hybrid Plasmonic Nanocircuits**

Junjun Shi,*^,1, 3^ Kangcheng Jing,^1, 2^ Li Li,^1, 2^ Wenjun Zhang,^2^ Tianzhu Zhang, *^, 2^ Xiaobo He^2^

^1^Shandong Provincial Engineering and Technical Center of Light Manipulations & Shandong Provincial Key Laboratory of Optics and Photonic Device, School of Physics and Electronics, Shandong Normal University, Jinan 250014, China

^2^Institute of Physics, Henan Academy of Sciences, Zhengzhou 450046, China;

^3^Henan Key Laboratory of Quantum Materials and Quantum Energy, School of Quantum Information Future Technology, Henan University, Kaifeng 475001, China

*E-mail: [jjshi@sdnu.edu.cn](mailto:jjshi@sdnu.edu.cn); zhangtianzhu@hnas.ac.cn

.

**S1. The thickness characterizations of the CdSe nanobelt**


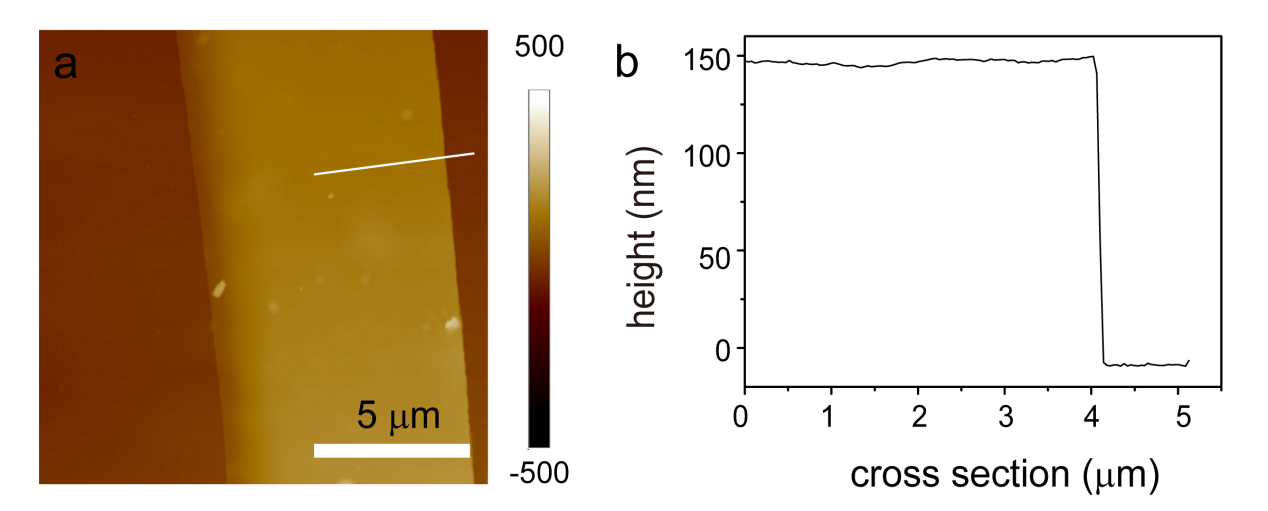


Figure S1. (a) AFM image of the nanobelt, where the scale bar is 5 μm. (b) Cross sectional profile showing the thickness of the CdSe nanobelt (~ 150 nm), extracted from the white line in panel a.

**S2. Parameter optimization for the CdSe NW**

To decrease the calculation load, we choose a straight waveguide with length 4 μm to calculate the SHG process. To allow comparison with the experiment, we excite SHG process in the simulations in a way similar to the experiments by illuminating one end of the wire with a circular polarized Gaussian beam. Figure S2 shows SHG energy flow as a function of the cdse nanobelt width. When the nanobelt width is increased, more modes are involved and their modes interaction increase the SHG efficiency. In this paper, we use the waveguide with width 500 nm as a proper choose.


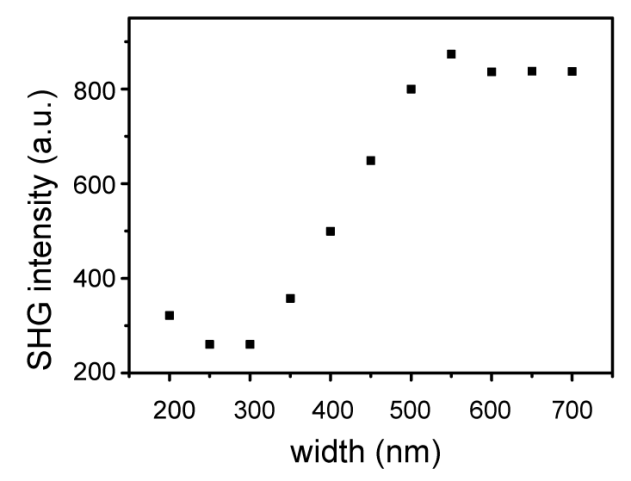


Figure S2. The SHG energy flow over the NW at different width.

**S3 The influence of the gap on the mode**

We analysis the effective index with enlarging the sweep range of the widths as shown in Figure 1. From the electric field distribution, we can find the energy of TM-like modes (TM0, TM1, TM2) is strongly confined to the Al_2_O_3_ layer. And these modes propagate mainly in the Al_2_O_3_ layer. We analysis the effective index as a function of the spacer thickness as shown in Figure S3a. The index of TM-like modes (TM0, TM1, TM2) decrease as the Al_2_O_3_ thickness increase. The electric field confinement property in the gap layer of these modes indicates that the gap layer influences the TM-like modes. The TE-like modes (TE0, TE1) store more electromagnetic energy in the nanowire from the electric field distribution and propagate mainly in the nanowire. The index of TE-like modes (TE0, TE1) slightly increases as the Al_2_O_3_ thickness increase. Figure S3b shows SHG energy flow as a function of the spacer thickness. The SHG intensity decreases when the thickness of the gap layer increased.


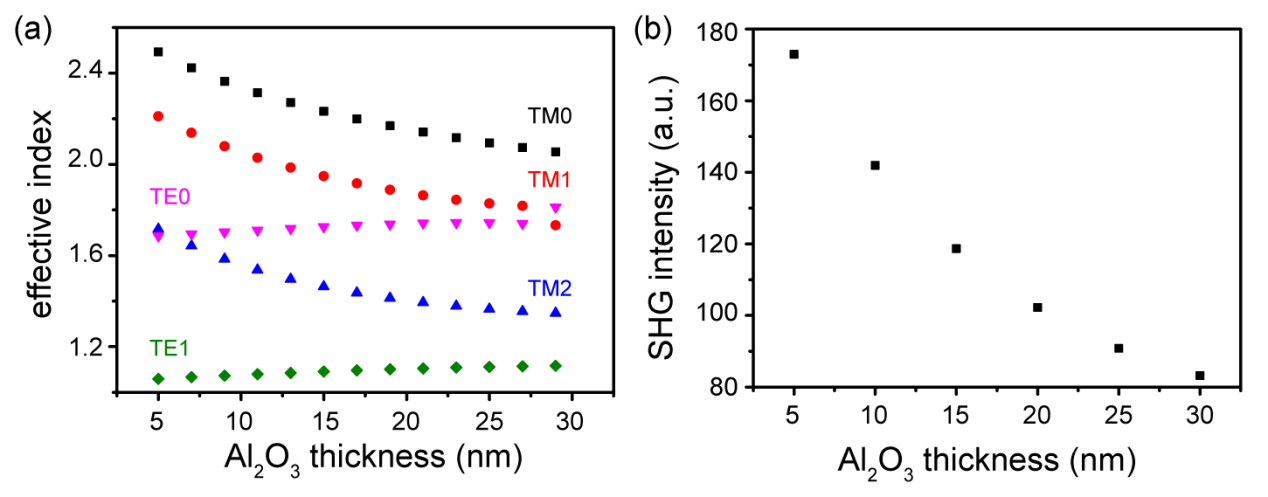


Figure S3. (a) The effective refractive index as the function of the width of the CdSe. (b) Calculated SHG energy flow over the NW as a function of the spacer thickness.

**S4 The near-field distribution in nanocircuit under different excitation**

To understand the origin of directional routing in Y-branch nanocircuit, we performed finite-element-method simulations using the commercial multiphysics software package COMSOL. To decrease the calculation load, we choose a straight waveguide with length 4 μm to calculate the SHG process. Figure S4a shows the zigzag electric field distribution by illuminating one end of the wire with a circular polarized focused Gaussian beam. To explore the origin the zigzag distribution, we used mode analysis and selectively launched the specific guided mode (s) into the simulated system. This method simplified the analysis of mode interactions. For example, one terminal was allowed to launch the excited modes simultaneously to model the counter-propagating modes and the other terminal use PMC boundary to reflect these modes. The simulated optical imaging at the fundamental frequency shows a similar periodic pattern along the NW as simulated using the Gaussian beam. The spin-selective routing for SHG can be understood using mode decomposition.


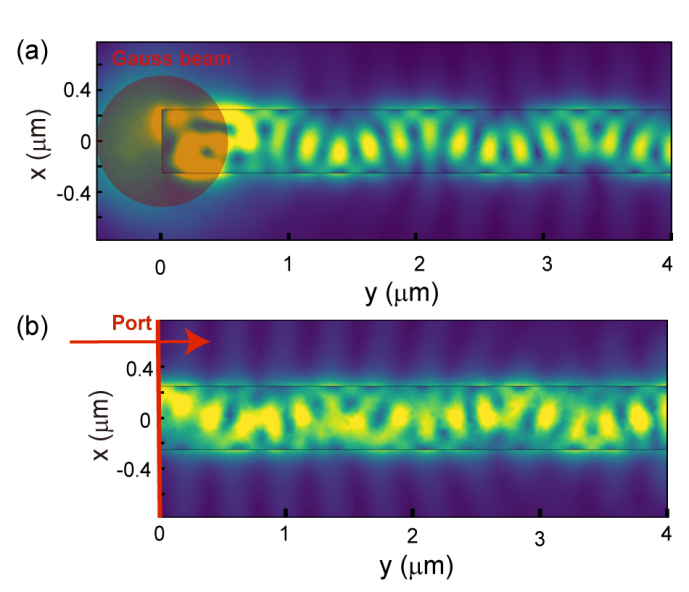


Figure S4. (a) Simulated electric field distribution at the center of the CdSe NW, at fundamental (800 nm) frequency. A circular polarized focused Gaussian beam is illuminating on one terminal of the NW. (b) Simulated electric field distribution at the same place using port input. The specific guided modes (TM0, TM1, TM2, TE0, TE1) into the simulated system.

**S5 The calculated far-field Fourier imaging of the SHG**


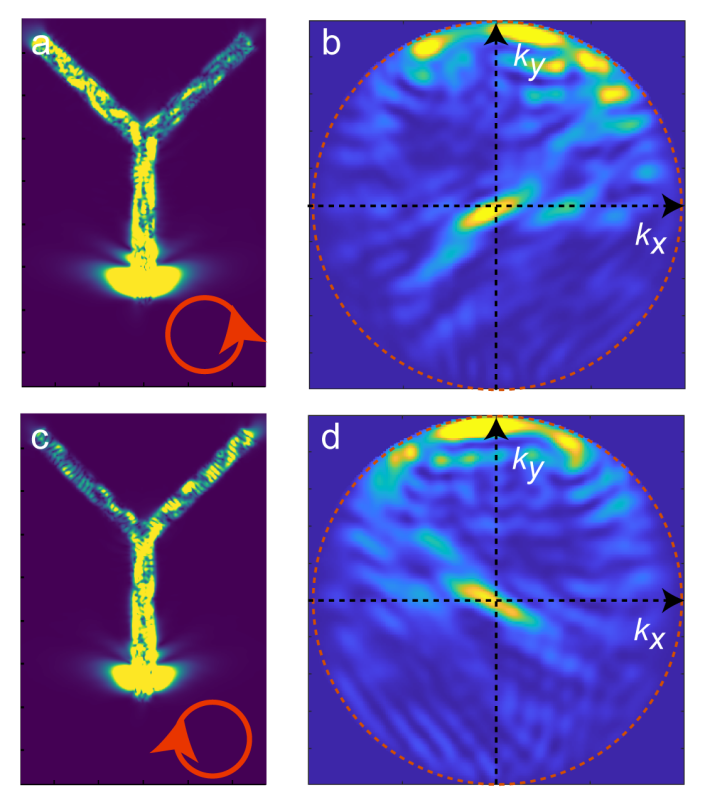


Figure S5. Simulated SHG in near field (a, c), and corresponding Fourier imaging (b, d), when the polarization is LCP (a, b) and RCP (c, d), respectively.

**S6. The evaluation of the SHG conversion efficiency**

To obtain the SHG conversion efficiency, we recorded the average power under the objective as *P*_FW1_ and obtained the integral counts *I*_1_ of the SHG image with an area size σ (the largest collection area of the spectrometer) from the whole Y-branch structure. After subtracting the SHG from the background with the same area size as *I*_2_, the SHG counts *I*_SH_ = *I*_1_ − *I*_2_ were obtained. The peak power P_FW_ was calculated taking account of the laser repetition rate and the laser pulse length (The laser repetition rate *ν* is 85 MHz and the laser pulse length *τ* under the objective is about 1 ps). So the peak power of incident light is *P*_FW_ = *P*_FW1_∕*ντ*. Then, we used a 400 nm laser to shine on the dielectric mirror (reflectivity close to 1) which reflected light through the beam splitter (transmission efficiency 50%), where the dichroic mirror is not suitable. We also recorded the integral counts *I*_3_ at the same area after subtracting the background counts, corresponding to the incident power *P*’_FW_ Finally, the SHG power from our sample is obtained as $P_{SH}'=\frac{I_{SH}}{I_{3}}$×*P*’_FW_×$\frac{50\%}{97\%}$ with a peak power of *P*_SH_ = *P*_SH_’∕*ντ*. The nonlinear conversion efficiency is *η*_SH_ =*P*_SH_∕$P_{FW}^{2}$.The nonlinear conversion efficiency is obtained about 3×10^-6^W^-1^.

**S7 The comparison of the routing ratio in different works**

| **Structure** | **Routing ratio** | **Signal** | **Reference** |
| --- | --- | --- | --- |
| Y-branched slot plasmonic waveguide | 14.5 db | laser | Nano Lett. 2019, 19, 1166.1171 |
| Y-branched Plasmonic waveguide | 14.8db | SHG | Nat. Commun. 2024, 15 (1), 1855 |
| Plasmonic Nanocircuits with Panda-Patterned Transporters | 0.95 | laser | ACS Photonics 9, 3089-3093 (2022) |
| TiO_2_ waveguide | 0.98 | PL | Nat. Nanotechnol. 17, 1178-1182 (2022). |
| branched Plasmonic waveguide | 13.24db | laser | Nano Lett. 20, 7543-7549 (2020) |
| branched slot plasmonic waveguide | 0.815 | laser | Nano Lett. 19, 3364-3369 (2019). |
| Y-branched Plasmonic waveguide | 0.857 | laser | Phys. Rev. Lett. 117, 166803 (2016) |
| Ag Nanowire | 0.915 | Raman | Phys. Rev. Lett. 123, 183903 (2019). |
| **Y-branched hybrid Plasmonic waveguide** | **0.767;**  **0.811** | **laser;**  **SHG** | **Our work** |

Table S1 The comparison of the routing ratio under different structures in different works
